# Abstract concepts: external influences, internal constraints, and methodological issues

**DOI:** 10.1007/s00426-022-01698-4

**Published:** 2022-07-04

**Authors:** Anna M. Borghi, Samuel Shaki, Martin H. Fischer

**Affiliations:** 1 Department of Dynamic and Clinical Psychology, and Health Studies, Sapienza University of Rome, 00185 Rome, Italy; 2 Institute of Cognitive Sciences and Technologies, Italian National Research Council, 00185 Rome, Italy; 3 Department of Behavioral Sciences, Ariel University, 44837 Ariel, Israel; 4 University of Potsdam, D-14476 Potsdam, Germany

## Abstract

There is a longstanding and widely held misconception about the relative remoteness of abstract concepts from concrete experiences. This review examines the current evidence for external influences and internal constraints on the processing, representation, and use of abstract concepts, like *truth, friendship,* and *number*. We highlight the theoretical benefit of distinguishing between grounded and embodied cognition and then ask which roles do perception, action, language, and social interaction play in acquiring, representing and using abstract concepts. By reviewing several studies, we show that they are, against the accepted definition, not detached from perception and action. Focussing on magnitude-related concepts, we also discuss evidence for cultural influences on abstract knowledge and explore how internal processes such as inner speech, metacognition, and inner bodily signals (interoception) influence the acquisition and retrieval of abstract knowledge. Finally, we discuss some methodological developments. Specifically, we focus on the importance of studies that investigate the time course of conceptual processing and we argue that, because of the paramount role of sociality for abstract concepts, new methods are necessary to study concepts in interactive situations. We conclude that bodily, linguistic, and social constraints provide important theoretical limitations for our theories of conceptual knowledge.

## Introduction

How do we know what we know? This longstanding and profound epistemological question has repeatedly been addressed, by a Cartesian search for firm foundations and by considering nativist against empirical evidence. The present inquiry is part of a Special Issue devoted to a small aspect of this question, namely our ability to distinguish between different formats of the mental representation of our knowledge. The special distinction at issue is between two types of conceptual knowledge, often referred to as “abstract” and “concrete”. We scrutinise this distinction without even knowing a proper and agreed-upon definition of concepts. Instead of belabouring this handicap further (see Murphy, 2004, and the many cogent introductory remarks in the contributions to this Special Issue for more reflections), we will bypass it here to find answers to our specific query through the study of human performance patterns. Our aim is to point to hitherto neglected clues that emerge from bodily and social constraints over our acquisition and representation of abstract knowledge.

### Abstract concepts and their varieties

Abstract concepts are often defined by what they are not (a definition by negation). For example, Google defines freedom as “the state of not being imprisoned or enslaved” and opinion as “a view or judgement formed about something, not necessarily based on fact or knowledge” (Google searches on 6. October 2021). Abstract concepts also tend to be dissociated from sensory properties that are directly related to experiences such as touch, taste, hearing, or smell (Barsalou et al., 2003), as indicated by property association tasks. Moreover, abstract concepts are often less iconic than concrete concepts (Winter et al., 2017). As a result of such observations, it might appear that abstract concepts lack an objective or shared mental representation that can easily be retrieved from memory. Such an impression receives support from their systematic cognitive processing disadvantages when compared to concrete concepts (Paivio, 1986). Importantly, abstract concepts are also typically low-dimensional, i.e. they do not share many common elements (Langland-Hassan et al., 2021; Lupyan & Mirman, 2013). Therefore, mental representations of abstract concepts can also be quite different between individuals and cultures (Borghi & Binkofski, 2014; Wang & Bi, 2021), subject to specific ways of acquisition, and context-dependent in their use (Falandays & Spivey, 2019). Finally, they also have some morphological specificities: for example, English mass nouns are considered more abstract than count nouns (Lievers et al., 2021).

Note, however, that concrete solid objects, such as tables or dogs, also include a huge variability across instances with regard to their perceptual or motor characteristics (think of a Great Dane in comparison to a Shi Tzu). Therefore, even concrete solid objects require a certain level of abstraction as part of the cognitive process of 'converting' them into their associated concrete concepts. Hence, instead of making a strictly dichotomous distinction, it is widely accepted today that all concepts are located on a representational continuum that ranges from more concrete (for those related to perceptible objects) to more abstract (for those with no tangible referent; cf. Barsalou et al., 2018). More recent views intend different kinds of concrete and abstract concepts as points in a multidimensional space (e.g., Borghi, 2022; Crutch et al., 2013; Harpaintner et al., 2018; Villani et al., 2019; Villani, Lugli, et al., 2021a). As a result, various types of concrete and abstract concepts can be defined according to their features and contexts (Barsalou & Wiemer-Hastings, 2005; Desai et al., 2018; Hampton, 1981; Caramelli & Setti, 2005; see Borghi et al., 2018a, 2018b; Villani et al., 2019,2021a, 2021b). This is an important theoretical change of perspective.

Crucially, abstract concepts come in different varieties. Surprisingly, while studies on concrete concepts have a longstanding tradition of research on the differences between categories, such as living and nonliving (Warrington & Shallice, 1984), or artefacts and natural objects (Keil, 1992), for years, abstract concepts have been considered as a unitary whole. One of the most interesting recent advancements in this research area has consisted in establishing that different kinds of abstract concepts exist (Borghi et al., 2018a; Desai et al., 2018; Muraki et al., 2020; for review, see Conca et al., 2021). In their recent review, Conca et al. (2021) examined 40 studies on kinds of abstract concepts, published until 2020. Across these studies, they found that the four kinds that recurred most often were emotional concepts, mental states ones, social and numerical concepts. The latter are typically investigated in isolation and rarely in relation to other concepts.

In some studies, sub-kinds of abstract concepts are defined a priori by the authors (e.g., Desai et al., 2018; Roversi et al., 2010; Caramelli & Setti, 2005), while in others they emerged from the data, reflecting either ratings or produced features (e.g., Crutch et al., 2013; Harpaintner et al., 2018, 2020; Villani et al, 2019). Even if they have overlapping aspects, these concepts differ in terms of the dimensions they elicit. A good illustration for this point is a study by Villani et al. (2019) where participants rated 425 Italian abstract concepts on 15 different dimensions, including perceptual strength, interoception, and involvement of hand and mouth effectors. Villani et al. (2019) identified three major components through Principal Component Analysis—a sensorimotor one, given by high ratings in perceptual strength (five senses) and hand involvement; an inner grounding one, characterised by higher ratings of interoception, metacognition, emotionality, sociality and mouth involvement; and an abstractness/concreteness one.

A further Cluster Analysis of these data set led to the identification of four clusters of abstract concepts which differed in their weights on four different components: Physical Space–Time and Quantity concepts, PSTQ (e.g., acceleration, effort) scored high on the sensorimotor and concreteness component; on the opposite side, Philosophical-Spiritual concepts (e.g., value, belief) scored low on the concreteness component. The third and fourth concept kinds, Emotions-inner states (e.g., anger) and Self and Sociality (e.g., kindness) had high inner grounding scores; Self and Sociality concepts scored higher than the other concepts also on their sensorimotor component.

As we can see, people can represent these different kinds of abstract concepts as points in a multidimensional space, characterised by dimensions with different weights. In a similar vein, Harpaintner et al., (2018) found with a property generation task that abstract concepts can be distinguished by their weights of specific semantic features, namely verbal associations, internal/emotional features, and sensorimotor features. In a meta-analysis in which abstract concepts were defined a priori, Desai et al. (2018) investigated the brain representation of numerical, emotional, morality, and theory of mind abstract concepts. They found some commonly activated areas, but also areas that were uniquely activated for a given kind of concept. Interestingly, abstract concepts differ also as to the effector they engage: numerical concepts typically activate the hand motor system, probably because of finger counting habits (Fischer & Brugger, 2011; Fischer & Shaki, 2018), while the mouth motor system is engaged to a larger extent by mental state concepts (Dreyer & Pulvermueller, 2018; Ghio et al., 2013). Finally, emotional concepts tend to activate those face and bodily regions through which emotions are expressed (Ghio et al., 2013; Moseley et al., 2012).

### The challenge of abstract concepts

All contributors to the present special issue agree that theories of concept acquisition and conceptual representation face a great challenge when it comes to abstract concepts. The argument goes like this: instances of concrete concepts share perceptual features that can be sensorially or motorically experienced and that may be part of the concept acquisition process. This learning experience may also be the starting point for generating and representing object prototypes. In contrast, instances of abstract concepts are intangible in nature, so it seems implausible that the same cognitive processes of acquisition and representation apply to both concrete and abstract concepts. However, this argument requires further scrutinization: A special and discriminatory role of sensory and motor processes can very well be attributed to seemingly abstract concepts. For example, we will below identify concrete sensory constraints during the acquisition of abstract concepts in the domain of numerical knowledge.

The argument of differential acquisition and representation for concrete versus abstract concepts has shaped the debate about the cognitive status of abstract concepts for a long time. Indeed, the ongoing debate still offers variants of the classical theories, such as Context Availability Theory (Schwanenflugel et al., 1988) or Dual Coding Theory (Paivio, 1986), extensions related to the Embodied Cognition approach (Barsalou, 1999, 2012; Glenberg & Kaschak, 2003), as well as more recent Multiple Dimensions Theories. These latter theories underline the importance of different representational dimensions characterising abstract concepts, ranging from affective aspects (Kousta et al., 2011; Newcombe et al., 2012; Ponari et al., 2018; Vigliocco et al., 2014) to interoception (Connell et al., 2018; Monti et al., 2021, this issue) and to language (Borghi, 2020; Dove, 2020). Among the theories of concepts that focus on language, some stress the role of language as neuroenhancements for conceptual representation (e.g., Language is an Embodied Neuroenhancement and Scaffold, LENS: Dove, 2014, 2020), others focus on the strict link between language and social interaction in the acquisition and representation of abstract concepts (e.g., Words As social Tools, WAT: Borghi & Binkofski, 2014; Borghi & Cimatti, 2009; Borghi et al., 2019—for a recent review on theories of abstract concepts see Borghi et al., 2017).

Given this currently active and intricate debate, the aim of the current review is to examine the up-to-date evidence of various external influences, as well as internal constraints, on abstract concepts. In Part 1: external influences, we ask: which roles do external influences such as perception, action, language, and sociality play in acquiring abstract concepts (that are, by traditional definition, detached from perception and action) and in conceptual development? What is the evidence for cross-cultural constraints on abstract knowledge? In Part 2: internal influences, we are interested in exploring how internal processes such as inner speech, metacognition, and bodily signals influence the acquisition and retrieval of abstract knowledge. Finally, in Part 3: some methodological considerations, we will also discuss some methodological issues regarding the time course of abstract concept acquisition and activation, recent insights in the domain of numerical cognition, and the role of novel, interactive methods. Before addressing how concrete constraints, both external and internal, influence abstract conceptual representations, we introduce a distinction between grounded and embodied cognition and briefly illustrate how embodiment and grounding can reveal concrete constraints on abstract concepts.

### Grounding and embodiment reveal concrete constraints

How do people understand abstract concepts, such as numbers? For example, is their understanding grounded in the use of fingers during counting, or are fingers perhaps an example of embodied cognition? In the literature on embodied cognition, the terms “grounded” and “embodied” are often used interchangeably, with other related terms (e.g., extended, enactive, embedded, situated) being closely associated (e.g., Newen et al., 2018). While this terminological confusion has been lamented before (e.g., Fischer, 2012), it has meanwhile become evident that a systematic distinction and application of terms can inspire specific research hypotheses about underlying cognitive mechanisms that generate the relevant behavioural signatures (see also Körner et al., 2022). Moreover, a clear differentiation between grounding and embodiment can help us to identify different types of concrete constraints on cognition. Given these benefits, we briefly explain the distinction here (cf. Pezzulo et al., 2013; Myachykov et al., 2014; for an opposing view, see Barsalou, 2020).

First, consider grounding as a fundamental constraint on the range of our cognitive activities. The human body is a physical object that has been exposed to and shaped by natural laws, including physical forces such as gravity and radiation, as well as biological forces such as sexual selection and heritability. Consequently, humans currently have an upright posture with muscles to withstand gravity and they possess five fingers on each hand to support object manipulation in the service of survival. Our sensory receptors are tuned to particular energy bands, with vision from around 400–700 nm, audition from about 20 to 20,000 Hz, and so forth (e.g., Wolfe et al., 2015). These evolutionarily inherited bodily configurations impose concrete constraints that ground our cognition in the real world.

A few sensory and motor examples serve to illustrate this idea: Grounding constraints govern our ability to detect stimulus changes, with sensitivity characterised by the Weber law in all modalities. Object individuation and tracking abilities establish signature limitations on human cognition, such as the ability to perform simple “calculations” a few months after birth (Wynn, 1992). Similarly, we expect light sources to be above us (e.g., Liu & Todd, 2004) and we orient towards the source of stimulation (e.g., Sokolov, 1960), abilities that support our precocious orientation relative to surfaces (Spelke, 2011). We anticipate objects to accumulate on top of each other instead of permeating into one another, giving rise to the universal “more is up” heuristic reflected in language metaphors (e.g., Lakoff & Johnson, 1980, p. 16). On the motor side, our body movements follow lawful constraints such as the two-third power law that relates angular speed of movement to trajectory curvature (Lacquaniti et al., 1983) or Fitts’s law that predicts our movement time from the size and distance of our action target (Fitts, 1954). The Fitts law, in turn, governs our perceptual appreciation of others’ motor capabilities (Grosjean et al., 2007). More generally, grounding enables domain-specific, encapsulated and automatic feats of cognition—so-called core systems of knowledge that reflect neuronal specialisations (Spelke & Kinzler, 2007).

While grounding of cognition reflects universal capacities of our evolved sensory and motor apparatus, embodiment of cognition refers to the result of idiosyncratic experiences with this apparatus. Specifically, our introductory example of finger counting can lead to a spatial association of small numbers with either left or right space, depending on how a person prefers to start counting on her hand (Fischer, 2008). More generally, cognition is embodied through an individual’s perceptual and motor history. It is unquestionable that sensory experiences differ as a result of genetic predispositions, thus rendering 8% of the male population colour blind (Wolfe et al., 2015, p. 143). But recent sensory-motor experiences also affect perception, as illustrated by the colour after-effect from wearing vertically split glasses with different tints (Bompas & O’Regan, 2006). Similarly, actions alter our categorization of shapes, such that our movement direction influences the perceived main axis of an object (Smith, 2005). Actions even influence our association of valence with space, such that left- and right-handers consider either left or right to be their good side, respectively (Casasanto, 2009). The idea of embodied influences on cognition will be illustrated further with reference to culturally mediated constraints (see below).

Before doing so, it is important to clarify the hierarchical relationship between grounding and embodiment with a perceptual and an action example. With regard to perception, our appreciation of visual quantity, for example the numerosity of a dot cloud, reflects automatic sensory integration across multiple visual features such as area, brightness, circumference, and convex hull (e.g., Clarke & Beck, 2021). Their discriminability is grounded in Weber’s law that gives rise to size and distance effects. Instead, number symbols are cultural developments that can replace the immediate sensory quantities across contexts and enable fine discrimination of large quantities; yet their understanding cannot escape the underlying sensory signatures, exhibiting size and distance effects, too. Symbolic notations with base-5 and base-10 number systems reflect both grounded and embodied constraints, namely the evolutionarily inherited structure of our body and also our cultural training. With regard to action, action opportunities depend on our body size, which grounds us in our multi-modal environment in the Gibsonian sense of affordances (Gibson, 1979). For example, specific experiences differ for short vs. tall people, resulting in embodied stair climb-ability judgments (Warren, 1984; Footnote 1). Finally, returning to finger counting once more, it should be clear that the shaping of abstract concepts reflects not either grounding or embodiment but their interplay, as well as situated constraints arising, for example, in the specific communicative context (Wasner et al., 2014). With these remarks, we are ready to describe how embodiment provides concrete constraints on cognition.

## Part 1: external influences

In the introduction, we have seen that different kinds of abstract concepts exist. We begin this first section of our review by providing evidence for the variability of abstract concepts and their resulting sensitivity to a range of external influences.

### Sensorimotor grounding with feature production tasks

Given the above evidence for different types of abstract concepts, it is not surprising that some types are in fact shaped by sensorimotor experiences to a larger extent than others. Evidence indicates that sensorimotor aspects are particularly crucial for some kinds of abstract concepts. For example, Villani et al. (2019) found with a rating task that they characterise mostly physical, spatial, temporal, and quantitative abstract concepts; and Harpaintner et al. (2018) found that abstract concepts were organised in different clusters. For one of these clusters, which included concepts like “observation” and “insight”, sensorimotor properties were dominant.

At a more general level, a variety of behavioural studies have highlighted the role of sensorimotor grounding for abstract concepts. Particularly informative are studies making use of feature production tasks. Feature production tasks, also called feature generation or feature listing tasks, consist of asking people either to freely produce words associated with the target concepts (word associations) or to generate the properties that are typically true of the concepts. They have been extensively used to assess conceptual representation (Wu & Barsalou, 2009) and allow to capture the conceptual relations elicited by different kinds of words by examining the produced features.

In one of the seminal studies on abstract concept representation, conducted with a feature generation task, Barsalou and Wiemer-Hastings (2005) had participants generate features for concrete, abstract and intermediate concepts (e.g., bird vs. invention vs. cooking). While both concrete and abstract concepts led participants to produce situations, the way these situations were characterised differed depending on conceptual abstractness. Abstract concepts yielded more features related to people, communication, and social institutions, and more extensive production of introspective features, while concrete concepts elicited more properties of entities. The results led the authors to propose that, while both concrete and abstract concepts are grounded in situations, for abstract concepts, the social aspects of situations are particularly crucial.

Harpaintner et al. (2018) obtained features for 296 German abstract words. They asked participants to generate and write down properties of situations coming to their mind when thinking about the word, and to write down four properties. The produced properties were then coded into 11 categories: sensorimotor ones (including the five senses and interoception), social constellations, internal states and emotions, associations, and other abstract concepts (e.g., karma as an association for sympathy). As to the results, we focus here on the sensorimotor features, which are more relevant for this review of external constraints. These were the most often produced properties (with a higher frequency of visual, motor-related, and acoustic features), followed by internal states and social constellations. However, sensorimotor features were highly variable across categories: Hierarchical cluster analysis revealed that abstract concepts are heterogeneous rather than indistinct categories. For example, in cluster analysis 1, based on the features sensorimotor properties, internal states, social constellations, and associations, the largest cluster was mainly characterised by sensorimotor features (e.g., observation, fitness); in a second cluster internal properties, in a third cluster verbal associations (e.g., present, theory) were in the foreground (e.g., nightmare, criticism). This study is important because it reveals that, although social and internal aspects are important for abstract concepts, sensorimotor features are dominant, even if their role varies greatly across concepts. Similar results were obtained by Banks and Connell (2021) who demonstrated with a category production task that perceptual strength (across the different perceptual modalities) of abstract concepts is quite high, particularly for some concepts (e.g., sport), even if still lower than that of concrete concepts.

Relevant for the present issue are also studies that focus on specific abstract concepts, such as gender or number. Mazzuca et al. (2020), (2021a), (2021b) asked Italian, Dutch, and English participants to list 10 words related to the word *gender*. Dutch and Italian cultures differ because of the more liberal attitude of the Dutch culture towards gender-sensitive topics, and Dutch and Italian languages differ also in terms of the gender-related pronouns used, which are two for Italian and three for Dutch. Consistently, Dutch people produced more concrete associations, linked to sensorimotor aspects and body parts (e.g., hormones, breast, genitals), whereas Italian participants used more words on the sociocultural dimension (e.g., discrimination, politics, power). This study reveals that the role of concrete determinants in representing abstract concepts can vary depending on the culture—here the same concept, gender, is conceived and grounded in more or less abstract features depending on the more or less liberal cultural attitudes towards gender.

Number concepts constitute another conceptual domain that illustrates the relevance of sensorimotor experiences in the shaping of abstract knowledge. In conflict with traditional beliefs about numbers as pinnacles of abstraction, both neuroscientific and behavioural studies show bi-directional links between bodily and numerical performance. As one would expect after a life-long history of finger counting, presenting small numbers activates parts of the same neural structures in the adult brain that control finger movements (Tschentscher et al., 2012). Similarly, both perceiving and performing finger counting postures systematically influence number processing speed but, importantly, only canonical finger postures (those people habitually adopt to communicate quantities) have the capacity to activate number knowledge (Di Luca & Pesenti, 2008; Sixtus et al., 2018).

Aside from sensorimotor studies, other rating studies indicate that abstract concepts involve effectors such as the hand and arm or the mouth and face. Ghio et al. (2013) found that abstract sentences referring to mental states involve both hand and mouth actions, presumably because these states are typically expressed by (inner or overtly) speaking and gesturing. In a similar vein, Granito et al. (2015) collected ratings showing that both the hand and the mouth were involved in actions with abstract concepts, while the hand was more involved than the mouth in actions with concrete concepts, independent of whether their members were very similar to each other or heterogeneous (for evidence on the role of mouth actions in abstract concepts acquisition, see Reggin et al., (2021)).

### Linguistic experience and abstract concepts

Many studies have emphasised the important role of language for abstract concepts. Before discussing the relationship between language and abstract concepts, it is noteworthy to say that recent literature suggests that language might be especially critical for some kinds of abstract concepts. For example, Villani, D'Ascenzo, et al. (2021) found with a rating task that language is particularly crucial for abstract institutional concepts (e.g., democracy), which are acquired later and through language rather than through perception. Harpaintner et al. (2018) found that words like “theory” and “dignity” formed a small cluster, where verbal associations played a major role.

Despite the centrality of language for abstract concepts, over the years, the authors have highlighted different aspects of language that might be relevant for their representation and use. The classical syntactic bootstrapping hypothesis (e.g., Gleitman et al., 2005) proposes that syntax leads children in the acquisition of word meanings and that they have an innate knowledge of the relationship between syntax and semantics. Hence, mastering many words and syntax is crucial to infer the meaning of concepts and to learn the so-called “hard words”, i.e. the more abstract ones.

Different from this theory, which focuses on the interrelationship between semantics and syntax, much recent research has been conducted in the framework of distributional semantics. This approach aims to capture word meanings by investigating the co-occurrence patterns of words in large corpora, effectively stating that word meaning is unrelated to sensory or motor experiences, regardless of whether the concept in question is concrete or abstract. Initially, proponents of strong embodiment theories criticized the capability of distributional theories to capture the gist of concepts without grounding the symbols in associated sensory or motor activity (Cangelosi et al., 2002), even if they could be useful instruments to assess linguistic regularities. They argued that the only way to capture meaning would consist in indexing words to real objects, agents, and situations (Glenberg & Robertson, 2000). In this framework, words played a rather peripheral role in that they were considered mainly as pointers to their referents. Recently, proponents of grounded theories are starting to recognize the important role that statistical linguistic information, derived from our experience with language, might play. In this perspective, some authors have proposed that words are shortcuts to meaning and might facilitate access to simulation (Barsalou et al., 2008; Connell, 2019; Connell & Lynott, 2013).

In contrast to the linguistic perspective on meaning representation and communication, the so-called simulation view argues that language can trigger inferential processes without explicitly stating the relevant concepts. This idea is nicely illustrated with the influential sentence-picture verification task which requires participants, in each trial, to first read (or listen to) a sentence and then classify a subsequently presented picture. Their task is to decide if that picture shows a previously mentioned concept. “Yes” decisions are faster when the object is depicted in the implied view (e.g., a bird with its wings spread rather than folded, when the sentence was “a bird flies in the sky”; cf. Ostarek & Huettig, 2019; Zwaan, 2014). While this method supports the notion of perceptual simulations in language comprehension, it is naturally limited to examining concrete (depictable) concepts.

### Theories highlighting the role of language: LENS and WAT

The role that linguistic and simulation information might play for conceptual knowledge activation is, however, debated. For example, Barsalou (1999, 2020) assigns a central role to simulation, arguing that mere linguistic information might only be sufficient to perform well in superficial linguistic tasks. In Barsalou’s view, once a person recognizes a word, associated linguistic forms are activated and can be used to respond to superficial linguistic tasks such as lexical decisions. These linguistic forms work then as pointers to simulations that involve deep processing and allow extraction of meaning. On the other extreme, Louwerse (2011) tends to believe that linguistic information carries most conceptual information, while other authors have an intermediate position, contending that both linguistic and sensorimotor experiences contribute to conceptual representation and are further modulated by the context and task (e.g., Connell, 2019).

The debate on the respective role of language and simulation is crucial if one considers the contrast between concrete and abstract concepts. Some recent proposals, such as the LENS (Language is an Embodied Neuroenhancement and Scaffold) and the WAT (Words As social Tools) proposals (Borghi, 2020; Borghi et al., 2019; Dove, 2020; Dove et al., 2020) have highlighted the role that language (both overt language and inner speech) plays for concepts in general, and particularly for more abstract ones. Because abstract concepts are low-dimensional, i.e. their members do not have much in common (Lupyan& Mirman, 2013), and because they are more detached from perceptual modalities than concrete concepts (Barsalou, 2003), language plays a critical role for them (Lupyan & Winter, 2018): labels represent a sort of glue that keeps heterogeneous category members together (Borghi & Binkofski, 2014). Consistent with this view, abstract words are typically acquired later in life than concrete ones and through language rather than through perception, as revealed by ratings on Age of Acquisition (AoA) and Modality of Acquisition (MoA) (Della Rosa et al., 2010; Wauters et al., 2003). Importantly, while LENS and WAT recognize the importance of both embodied experiences and linguistic information to construct and access meaning, the WAT theory in particular highlights two aspects, the involvement of the mouth motor system during abstract conceptual processing and the strict interrelation between linguistic and social dimensions. We elaborate and explain these two points next.

### WAT, mouth activation, and social interaction

Evidence obtained with different tasks, such as ratings (e.g., Ghio et al., 2013), behavioural tasks with children and adults (review in Mazzuca et al., 2021a, 2021b), and fMRI (Dreyer & Pulvermueller, 2018) has revealed that the mouth might play a critical role in abstract conceptual processing. Despite some inconsistent results (for example in rather shallow semantic tasks like lexical decision, e.g., Mazzuca et al., 2018), mouth responses are typically facilitated during abstract but not during concrete concept processing. Moreover, actively moving the mouth, for example, while chewing gum, interferes less with the processing of concrete than abstract concepts (Villani et al., 2021a). Even though it still needs to be determined whether the involvement of the mouth is necessary to access the meaning of abstract concepts, these observations certainly testify to the strict interrelation between semantics and phonology, and the importance of language for abstract concepts.

The second aspect underlined by WAT is the importance of the social dimension for abstract concepts. Developmental evidence reveals the importance of social abilities for abstract concept acquisition. In a study by Bergelson and Swingley (2013) parents named events displayed in videos to their children; children successfully looked at events related to abstract concepts by 10 months, with a marked increase at 14 months. Notably, the increased processing ability corresponded with the acquisition of important social abilities, i.e., the ability to follow other people’s gaze and to engage in joint action with others. This evidence suggests that social abilities are critical for the acquisition of the first abstract concepts and fosters the view that social interaction is more crucial for their acquisition than for the acquisition of concrete concepts.

Not only abstract concepts are grounded in social interaction; their use might also foster group cohesion. Influential work in social psychology revealed the Linguistic Intergroup Bias (LIB, Maas et al., 1989): When referring to your in-group, you tend to describe positive behaviours more abstractly than negative ones (e.g., X is helpful rather than X helps), while the opposite is true for the outgroup (e.g. X is aggressive rather than X hurts others). More recently, Gilead et al., (2020) proposed that groups that define their beliefs in terms of abstract concepts might become more cohesive. For example, they claimed that a sentence like "I am a Republican because I believe in liberty", which involves an abstract concept, might lead to a higher social acceptance than a sentence like "I am a Republican because I don't want the government to take away my guns." This strategy makes statistical sense because the more abstract statement encompasses a larger group of opinions, compared to the concrete statement. Borghi et al. (2018b) have recently coined the term “social metacognition” to suggest that the cognitive difficulty of abstract concepts makes people more aware of their knowledge limitations and a need to revert to others for support and information (Shea, 2018). This mechanism, which is particularly powerful with abstract concepts, might foster social cohesion. Below we will discuss recent evidence showing that social metacognition might also impact motor actions towards other people.

### Concrete cultural constraints on cognition

As anticipated in the introduction, one crucial characteristic of abstract concepts is their variability within and across participants. Because their meaning is more open and undetermined, they are more likely to be influenced by their context. Consistently, classical studies on contextual availability show that concrete concepts are more linked to specific contexts, while abstract ones evoke heterogeneous contexts and situations (Schwanenflugel et al., 1992). In light of these considerations, it is crucial to understand how and to what extent the cultural context modulates conceptual meaning.

Culture itself is an abstract concept, similar to freedom or justice, because it has no immediate and agreed referent. Instead, what is generally referred to as culture is expressed and acquired through concrete interactions with objects or with people. For analytical purposes, these interactions can be classified into two broad subtypes: sensory-motor and social-linguistic interactions (cf. Borghi & Binkofski, 2014), both of which lead to embodied experiences. An interesting aspect of linguistic interactions is their ability to provide indirect embodiment: knowledge can be defined through other concepts without the need for sensory-motor enrichment. For details of this idea, see Körner et al. (2022). In this section, we review culturally mediated sensory-motor habits that exert concrete constraints on abstract concepts. However, to trace the possible mechanisms of such cultural shaping of cognition, we will also include more general findings of cultural influences on attention deployment, perceptual judgments, face processing, and aesthetic preferences.

### Visual scanning and spatial attention

Fine-grained analyses on the different kinds of abstract concepts reveal that space, time, and numerical concepts typically cluster together and activate dimensions differing from these elicited by emotional, social, philosophical, and mental state abstract concepts. Conca (2021) uses the term “magnitude” to refer to these concepts (see also Troche et al., 2014, 2017). Rating studies show that, compared to other abstract concepts, they obtain high scores in sensorimotor features (Villani et al., 2019) and the hand effector's involvement (Ghio et al., 2013). Consistently, neuroscientific studies reveal that these concepts typically engage the intraparietal sulcus (IPS), and the middle and frontal prefrontal gyrus, areas typically involved in processing of magnitude. Due to the critical role these concepts play, the next sections will be dedicated to addressing the sensorimotor determinants of these concepts—space, time, and quantity.

Considering sensory–motor interactions, perhaps the most obvious candidate for culturally constrained cognition is the fact that people learn to read and write text either from left-to-right (in Western cultures) or from right-to-left (for example in Arabic, Farsi, and Hebrew). The asymmetry of this spatial-motor habit has decisive perceptual and cognitive effects. Early findings already showed clear spatial biases for visual scanning in search for information: People begin their visual exploration of space from the top left (Brandt, 1945) and exhibit greater reproduction accuracy for pattern elements that were briefly presented to the left of our fixation (Anderson, 1946; Anderson & Crosland, 1933). Subsequent cross-cultural studies found different biases for left-to-right and right-to-left readers. For example, Abed (1991) presented symmetrical dot patterns to three groups of participants with different reading direction habits: left-to-right Westerners, right-to-left Middle Easterners, and a group of East-Asian participants who were experienced with top-to-bottom reading direction. Interestingly, participants from all groups fixated more on the top left than on the other portions of the dot patterns. However, significant differences between cultures were found with horizontal vs. vertical saccades, as well as left-to-right vs. right-to-left saccades, consistent with the reading direction of the participants’ reading direction. Specifically, both left-to-right and right-to-left readers had more horizontal than vertical saccades, while top-to-bottom readers had more vertical than horizontal saccades. Moreover, left-to-right readers had significantly higher left-to-right than right-to-left movements, while right-to-left readers showed the reverse pattern. Finally, top-to-bottom readers had similar left-to-right and right-to-left movements.

Similar influences of reading direction habits on eye movements were found in a series of experiments on visual exploration of images (Afsari et al., 2016). For example, bilingual participants first read left-to-right or right-to-left text, then explored images. Left-to-right readers who learned a right-to left language late in life had leftward shift after reading either left-to-right or right-to-left text. However, native right-to-left readers showed a leftward bias after reading left-to-right text, but rightward bias after reading right-to-left text. Reading direction habit influences also the selection of horizontally presented items for English and Arabic students (Ariel et al., 2011). People are also better at recalling information that contains ordinal stimuli if the spatial flow of presentation during encoding matches the directionality of their habitual reading (McCrink & Shaki, 2016).

These opposite cultural scanning habits already bias spatial search strategies of English and Hebrew speaking preschoolers aged 3–4 years (McCrink et al., 2014). The children were first introduced to two boxes, the sample box and the matching box, comprising five compartments each. The compartment search had a picture of a common object on it, with different order of pictures for each box. Then the experimenter labelled the compartments using the letters A–E, with identical or opposite horizontal direction of labels for the boxes. Finally, children learned a game of hide-and-seek with two monkeys, in which one monkey was inserted into a randomly chosen compartment in the sample box and they had to guess where the other monkey hid in the matching box. Children were better able to use these labels to complete a matching task when the compartments were verbally labelled in a congruent direction of their cultural reading direction. Clearly, the predominant exploration behaviour in a culture imposes concrete constraints on the cognitive signatures of its youngest members.

### Perceptual judgments

Following this substantial constraint on the direction of visual scanning of space as the result of reading direction habits, other spatial asymmetries between participants with opposite reading directions were also investigated in the context of simple perceptual judgments. For example, a left bias in the visuo-motor task of bisecting horizontal lines was found, in which participants typically mark the subjective middle to the left of the objective centre (for review see Jewell & McCourt, 2000). A series of studies by Chokron and colleagues (Chokron et al., 1998) found line bisection to depend upon participant’s reading habits. While the left bias was demonstrated in left-to-right readers, right-to-left readers bisected the lines to the right of their objective centre. This cultural constraint on our fundamental ability to perceptually evaluate simple stimuli was also found with other tasks, such as line extension (Chokron et al., ), line trisection and quadrisection (Zivotofsky, 2004), and computerised variations of line bisection (Nicholls et al., 2002; Rinaldi et al., 2014). Culturally mediated biases extend even to the bisection of words (Fischer, 1996; Gabay et al., 2015; see also Fagard & Dahmen, 2003), reflecting embodied constraints on how people handle abstract symbolic materials.

While the influence of everyday (active or passive) exposure to habitual reading habits on perception is well established, one should be aware of the surprising flexibility of this sensory-motor constraint on cognition. Rosenich et al., (2020) employed a simple mirror-reading task prior to spatial judgement of pre-bisected lines. As expected, left-to-right and right-to-left readers showed opposing spatial biases after reading standard text. However, only 20 min of mirror reading shifted the response bias (correspondence to the current direction of reading) compared to the standard reading. These findings indicate the importance of recent over long-term sensory-motor activities when it comes to their constraints on cognition.

Preferences depending on spatial position The abstract concept of beauty has inspired philosophical speculations since ancient times. The field of aesthetic preferences became one of the earliest branches of experimental psychology in 1876, when Fechner published his ‘Vorschule der Aesthetik’. When exploring asymmetrical images, various spatial biases exist. For example, a leftward posing bias is evident in painted portraits (e.g., McManus & Humphrey, 1973; Nicholls et al., 1999). A possible neurobiological explanation suggests preferential activation of the right hemisphere inducing an attentional bias to the contralateral left visual field (e.g. Loftus & Nicholls, 2012; Ossandón et al., 2014). Note, however, as we reviewed above, leftward perceptual and attentional biases diminished or even reversed when tested in right-to-left readers.

Are these aesthetic effects also sensitive to the reading directions of participants? Here we review cross-cultural studies that tested the influence of reading direction habits on various aesthetic preferences, thus identifying a concrete constraint on abstract judgments. Chokron and De Agostini (2000) investigated aesthetic preferences for mirror images with directional static and moving objects (e.g. leftward and rightward statue or truck). Left-to-right French readers indicated more frequently that the rightward image was more aesthetically pleasing, “in flow” with their direction of reading, while right-to-left Hebrew readers preferred leftward objects. Similar patterns were found in follow-up studies with ethic groups of various reading directions (Ishii et al., 2011; Nachshon et al., 1999; Nittono et al., 2020) as well as for dynamic video stimuli (Friedrich et al., 2014, 2016) and evaluation of fashion garments on the runway in left-to-right or right-to-left motion (Flath et al., 2019). When a series of objects are horizontally placed in a picture, left-to-right readers preferred compositions with the object of interest is to the right rather than on the left side of the composition (at the beginning of the habitual scanning direction (Christman & Pinger, 1997). However, Christman and Rally (2000) found a weaker effect for right-to-left readers. Moreover, in a follow-up study by Heath et al. (2005), left-to-right, right-to-left, biliterate readers, as well as a group of illiterates were asked to choose between pairs of pictures, each consisting of three geometric elements arranged laterally. As expected, aesthetic preferences depended on participants’ reading habits: They preferred to begin their scan with interesting objects (or either interesting or more salient objects in case of unbalanced composition) and moved in the direction consistent with their reading direction.

Reading direction influences not only the aesthetic preference for directional objects that “flow” with one’s scanning habits but also biases the perceived actions described in these pictures as more forceful or dynamic than the mirror objects. For example, Maass et al. (2007) found that the same action was interpreted as stronger, faster, and more beautiful if presented rightward for Italian left-to-right readers, but leftward for Arabic right-to-left readers (see review in Page et al., 2017). Interestingly, attribution of feature magnitude is also influenced by reading direction: When two fictitious products were spatially presented, English speakers tended to attribute greater features (e.g. powerful, refreshing or potent) to the left product, but the reverse pattern was obtained for Farsi speakers (Von Hecker et al., 2021).

Relatedly, not only perceived but also performed actions are shaped by reading habits. This is evident from finger counting habits in different cultures: While most Western countries prefer a left-hand start, Iranian participants predominantly prefer to begin counting on their right hand (Lindemann et al., 2011; Shaki et al., 2012). While other cultural rules and practices may contribute to this “spill-over”, it is also clear that observational learning is a powerful source of cultural transmission. This was illustrated for object counting direction in the work of Göbel et al. (2018) who reported that already 3–5 year-olds count a horizontal array of objects in the direction that they have just observed in the reading behaviour of a caregiver.

Reading direction habits also shape our mental representation of spatial relations between objects and spatial associations of abstract concepts. For example, Tversky et al. (1991) asked children to place stickers on square pieces of paper to represent relations between various concepts. They found that relations of temporal concepts were placed horizontally mostly from left-to-right for left-to-right English children, but the opposite direction was dominant for right-to-left Arabic children. Fuhrman and Boroditsky (2010) found similar opposite patterns between English and Hebrew speakers in a variety of tasks: arranging pictures depicting temporal sequences of natural events, pointing to the hypothesised location of events relative to a reference point, and rapidly deciding whether the second picture in a pair is conceptually earlier or later than the first picture. Moreover, even without any temporal or spatial relations between objects, American caregivers were more likely to place pictures in a story construction task from left-to-right than Israeli caregivers (McCrink et al., 2018).

### Spatial associations of abstract concepts—the case of numbers

As might be expected from the selected numerical cognition studies mentioned above, reading habits also influence our mental representation of numbers and how people manipulate them. A clue for this concrete constraint over abstract number concepts came from the landmark paper by Dehaene et al. (1993) which established systematic spatial-numerical associations: small numbers are associated with left space and larger numbers with right space (the SNARC effect; spatial-numerical association of response codes). This effect was documented in a parity judgement task with centrally presented digits and lateralized button responses. Importantly, the spatial association depended on cultural immersion: In Iranian participants, the association was stronger for those who had lived in Western culture for longer. Later work by Shaki et al. (2009) established this correlative dependency of SNARC on reading direction more clearly by documenting its absence in Israeli readers who read Hebrew text from right-to-left but numbers from left-to-right, as well as a complete reversal of SNARC in Arab readers who read both Persian script and numbers from right-to-left.

A causal dependency was documented in bilingual Hebrew-Russian readers who can be exposed to both left-to-right- and right-to-left orthographies. Shaki and Fischer (2008) instructed these bilinguals in separate blocks with either of these two languages and showed that the strength of their SNARC effect depended on the instruction language. Fischer et al. (2009) examined the time course of this mapping by randomly varying the language of number words between the Cyrillic and Hebrew script and recording the speed of parity judgments in bilingual readers. The SNARC effect was reliably smaller for Hebrew compared to Cyrillic number words. Importantly, each word was followed by a digit that also had to be evaluated by parity, thus assessing how visually identical materials were processed as a function of the reading direction in the previous trial. Surprisingly, the SNARC effect varied from one second to the next, changing consistently with the recent scanning direction. This result again illustrates that reading habits exert no strong constraint over the spatial representation of abstract concepts and can flexibly and rapidly adapt to the current situation, consistent with the results from line bisection after mirror reading (Rosenich et al. (2020, see also Roman et al., 2015).

A further study by Fischer et al. (2010) underlined this re-evaluation of the power of reading habits over spatial cognition by holding reading direction constant while manipulating the position of numbers on a page. Participants read 20 cooking recipes for a memory test where the quantity information contained in them was distributed either SNARC-congruently (all small numbers on the left, all large numbers on the right side of the page) or incongruently between groups. While the pre-test revealed identical SNARC effects in both groups, the incongruent group exhibited a significant reduction of SNARC after the manipulation. Interestingly, a repetition of this study with Hebrew participants replicated this effect by inducing a SNARC effect in the “incongruent group” (where number positions were mapped onto space congruently with their reading direction). Thus, without changing reading direction, it is possible to manipulate spatial-numerical mappings, indicating that location associations contribute to SNARC and thus provide a further concrete constraint over the cognitive representation of abstract concepts.

It has become clear that embodied constraints over conceptual representations reflect not only sensory-motor experiences but also inherited dispositions people share with other animals. Considering further the case study of numerical cognition, evidence for spatial-numerical associations in newborns and animals (Di Giorgio et al., 2019; Rugani et al., 2007) has long pointed to hemispheric pre-disposition for a left–right preference. While Rugani et al. (2007) postulated a right-hemispheric specialisation for number processing in all vertebrates, De Hevia et al. (2017) promoted an attentionally mediated scanning preference for increasing numerosities. A more recent account (Felisatti et al., 2020a, 2020b) identified the higher frequency-related filtering abilities of the left compared to the right brain hemisphere as a further candidate mechanism for this pervasive association of small quantities with left space. While further research is needed to resolve this issue, all proposals agree that there must be a grounded constraint on numerical cognition.

In summary, each aspect of culture, from the fundamental social factors to everyday sensory and motor factors, affects or reflects our perception and cognition. Moreover, the few experimental manipulations we have reviewed above indicate that at least some cultural habits create an inescapable constraint on the way people perceive the external environment and mentally represent it. These embodied constraints are often indicative of underlying grounded constraints.

## Part 2: internal influences

This second section of the review switches perspective and directs readers’ attention away from external influences that establish sensory and motor cues for the efficient processing of abstract as well as concrete concepts. Instead, we now focus on processing constraints that emerge from within the human body. The section begins with a review of rating studies before considering inner speech and interoceptive signals.

### Metacognition and inner speech for abstract concepts

How do internal processes such as inner speech, metacognition, or sensitivity to internal bodily signals, influence the acquisition and retrieval of abstract knowledge? Rating studies show that people think interoception and metacognition are more important for abstract than for concrete concepts (Connell et al., 2018; Villani et al., 2021b). Moreover, participants claim that they need others more to comprehend abstract compared to concrete concepts (Mazzuca et al., 2022), and particularly more abstract concept kinds (Villani et al., 2019). Notably, interoception rating scores are typically correlated with emotional ones (Mazzuca et al., 2022; Villani et al., 2019), and characterise mostly emotional abstract concepts (Villani et al., 2021a). According to one influential theory, the Affective Embodiment Account (Kousta et al., 2011; Vigliocco et al., 2014), abstract concepts are characterised more than concrete ones by affect and valence. However, in the literature, there are also contrasting results, showing with a lexical decision task that concrete rather than abstract concepts are more associated with valence (Yao et al., 2018) and it is currently debated whether emotionality and affectivity characterise all abstract concepts or mostly emotional and mental states concepts.

The complexity of abstract concepts has led some authors to propose that a metacognitive monitoring process might be of special relevance for these concepts, and this metacognitive process might lead us to defer to others (Borghi, 2022; Borghi et al., 2018a, 2018b; Shea, 2018). Specifically, metacognitive awareness of the limits of our knowledge in a given abstract domain might have two possible outcomes. First, it might induce people to perform an extended inner search for meaning; if this does not succeed, they might prepare to interact with others, either to ask them for information or to negotiate with them the meaning of the contentious concept (Borghi, 2022; Borghi et al., 2021). As argued by Shea (2018), such deference is often metacognitive, in that it concerns the belief about one’s own concepts. Hence, our metacognition exerts a concrete constraint that would be involved in monitoring one’s own concepts and in relying on others to improve them. Some authors have proposed that inner speech, i.e. talking to oneself without an audible counterpart (Alderson-Day & Fernyhough, 2015; Perrone-Bortolotti et al., 2018), might also play a role, both in the form of talking to oneself and of rehearsing previous conversations with others (Borghi, 2020; Dove et al., 2020). Inner speech might intervene both during our search for meaning and while preparing to ask others.

The idea that inner speech is useful to search for meaning is compatible with neural evidence. Studies showing a selective activation of the left Inferior frontal gyrus during abstract concept processing (meta-analysis in Binder et al., 2005; Della Rosa et al., 2018; Wang et al., 2010) are in keeping with the view that abstract concepts involve longer processing in phonological working memory (Binder et al., 2005). However, the specific role inner speech plays has to be elucidated by future research. So far, evidence is limited to a few studies. In a recent behavioural study, Fini et al. (2021a), (2021b) have shown that articulatory suppression (continuous pronunciation of a syllable), an activity typically used to disrupt inner speech, influences more the simultaneous categorization of abstract than of concrete concepts; the same is not true for an activity involving the hand, like ball squeezing (Fini et al., 2021a). However, the pattern is further complicated by the fact that, at least according to some authors, inner speech does not necessarily involve articulation; furthermore, so far the evidence produced is limited and not very strong (for reviews, see Langland-Hassan & Vicente, 2018; Lœvenbruck et al., 2018). Compatible neural evidence shows that during abstract thought, neural areas linked to inner speech are engaged (Berkovich-Ohana et al., 2020). There is also some evidence from autistic spectrum condition (ASC) individuals who scarcely make use of inner speech (Granato et al., 2021). ASC individuals have some impairments during categorization and prototype extraction (e.g., Church et al., 2015), but the evidence that they have more difficulties in processing of abstract compared to concrete concepts is scattered and controversial results are present (e.g., Eskes et al., 1990). Thus, inner speech may or may not shape our abstract thinking. In any case, it would be difficult to ascribe a processing problem with abstract concepts in ASC individuals solely to the absence or reduced use of inner speech.

### Interoception and abstract concepts

Aside from the five traditional senses that provide an external grounding of abstract knowledge through vision, audition, smell, taste and touch, one further sensory source is deemed as crucial for the representation and processing of abstract concepts. We refer to interoception, i.e. the multi-modal sensory afferents from within the body that emerge from our internal organs like the lungs, guts, joints and arteries. Berntson et al., (2019, p. 3) distinguish between visceral and somatic bodily afferents. Visceral afferents provide internal states of the organs, tissues and both olfactory and gustatory receptors. Somatic afferents include proprioceptive and tactile sensory input derived from our muscles, joints and skin. These are predominantly transduced via the vagus nerve and integrated in the anterior insula and other cortical centres (recent reviews in Azzalini et al., 2019; Berntson & Khalsa, 2021; Petzschner et al., 2021).

Studying the influence of bodily signals has revealed surprising modulations of cognitive performance signatures, as early as stimulus encoding: proprioceptively perceived body posture affects several supposedly normative visual attention signatures (e.g., search slopes, attentional blink, inhibition of return and flanker effect): these differ between a condition where visual stimuli are presented on a computer screen that is physically distant from the body, and an “embodied” condition where the display is held with the hands (Abrams et al., 2008; Davoli & Brockmole, 2012). Other well-known embodied cognition examples, more related to the issue of abstract knowledge and its accessibility, include the effects of facial muscle activity on emotional language comprehension, as reflected in impaired processing of emotional language after Botox injection (Havas et al., 2010) and on perceived stimulus valence (Strack et al., 1988; for an update see Noah et al., 2018). Interestingly, there seems to be a contribution from our blood sugar levels to our ability to make juridical decisions, with more lenient decisions made directly after food intake (Danziger et al., 2011). Finally, even the perception and production of numbers seems to be sensitive to interoceptive signals that emerge from our repetitive breathing, as was recently documented by Belli et al. (2021). More generally, attending to interoceptive signals from the body in cognitive assessments has generated surprising insights into the variability with which different people select and use this information.

In a recent rating study, Connell et al. (2018) demonstrated that interoception, called by the authors “the forgotten modality”, characterised more abstract than concrete concepts, and particularly emotional ones. Modifying a task typically used to assess interoceptive accuracy, Villani et al. (2021a), (2021b) asked participants to estimate their heartbeat pace and concurrently to evaluate the difficulty of different kinds of concrete and abstract words. Results showed that the perceived difficulty of abstract words increased during the heartbeat estimation task when compared to other conditions (ball squeezing, gum chewing, and articulatory suppression). This finding held in particular for emotional-social and philosophical-spiritual abstract concepts. Overall, these studies suggest that interoception might be critical for abstract concept representation, and particularly for the representation of emotional abstract concepts. Further work is needed to establish mechanisms for the integration of interoception into the processes of abstract concept formation and development.

## Part 3: some methodological considerations

We can see several methodological challenges to our understanding of abstract conceptual knowledge representation. These challenges reflect and enhance issues that are already familiar from the study of concrete concepts. Without any pretence of broad coverage, we will briefly discuss the importance of understanding the time course of conceptual activation and highlight a few new methodological approaches worthy of consideration.

### Time course

The time course of knowledge activation is hard to understand on the basis of outcome measures such as reaction times and accuracy rates. This is so because these measures conflate the durations and successes of many preceding cognitive operations. More highly time-resolved measurements are desirable; they traditionally include eye-tracking and electrical recordings of brain activity by means of electro-encephalography (EEG). More recently, the recording of movement trajectories from mouse cursor coordinates or from the contact points of the index finger sliding across a touch-sensitive screen has been added to this repertoire as more versatile and less demanding methods to obtain highly time- and space-resolved evidence about simultaneously unfolding cognitive processes. While mouse and finger tracking are both inspired by the rationale that embodied concepts should involve bodily movement signatures (Dijkstra et al., 2014; Felisatti & Fischer, 2023; but see Barsalou, 2020), they differ in their interpretability: Mouse trajectories involve an additional spatial transformation (usually from forward movements of the hand to vertical movements of the cursor) that may or may not reflect conceptual associations. Finger tracking provides more direct evidence in the sense that this coordinate transformation is not included in the response planning processes. More recently, the recording of spontaneous fluctuations in grip force seems to be a promising addition to the tool kit of researchers interested in establishing embodiment signatures (Nazir et al., 2016). In this method, participants merely hold a highly sensitive force sensor between their fingers and passively perceive conceptual information. An example would be to listen to a sentence like “John lifts the suitcase”, which induces a spontaneous increase in holding force that suggests motor simulation to be part of the sentence comprehension process. Moreover, negated sentences or counterfactuals do not induce this simulation signature. The fact that motor activation occurs spontaneously and is scaled with the effort implied in the stimulus provides highly time-resolved evidence in support of the semantic somatotopy model put forward by Pulvermüller and colleagues (e.g., Pulvermüller et al., 2021) and reflects biological constraints from the structure of the cortical surface. An extension of this methodology to the comprehension of abstract (numerical) concepts seems to be successful (Miklashevsky et al., 2021; Mikashevsky, 2022).

All such methods have in common that they are able to track the emergence of processing signatures related to the meaning of concepts, including abstract ones. An illustration of the benefit of methodological convergence is the work on the time course of spatial-numerical associations, which has been studied with eye tracking (Masson et al., 2018; Myachykov et al., 2015, 2016) as well as mouse or finger tracking (Marghetis et al., 2014; Dotan & Dehaene, 2017).

### Interactive methods

Most studies on abstract concepts make use of single words or simple sentences, adopting either feature generation or rating tasks, or tasks in which response times are recorded, such as lexical decision, and sentence sensibility evaluations. Only a few studies have so far adopted interactive methods, i.e. investigating abstract concepts and the words expressing them during their use. This work is quite diagnostic about the cognitive representation of abstract concepts because these tend to be more context-dependent in their meaning and use.

For example, Zdrazilova et al., (2018) asked pairs of participants to perform the so-called taboo game: one of them had to explain the meaning of the word to the confederate, without mentioning the word itself, and the other had to guess it. The authors analysed both the verbal productions and the gestures that were spontaneously generated. In the verbal productions, they found an increase of person-related and introspective features with abstract compared to concrete concepts (see Barsalou & Wiemer-Hastings, 2005, for a similar result on introspection). As to gestures, metaphoric gestures (e.g., moving the hands forward to speak of the future) and beat gestures (i.e., gestures having no specific meaning but accompanying the speech prosody) dominated with abstract concepts, while iconic gestures that represented physical objects or events were more frequent with concrete concepts. Thus, specific patterns of embodiment can be linked to specific concepts.

More recently, Fini et al., (2021a) performed an interactive kinematics task to compare concrete and abstract conceptual knowledge representations. The experiment consisted of two blocks, one focussing on abstract and the other on concrete concepts; for each block, there was a separate experimenter. The experimenter showed participants photos and asked them to guess the (concrete or abstract) concepts the images represented; participants could ask for help, and when required the experimenter gave them suggestions. Overall, participants perceived the task with abstract concepts as more difficult and asked for more hints. The guessing task was followed by an interactive joint action task, in which each participant had to grasp a bottle together with an avatar which embodied the experimenter participants had previously met. Participants were told that, after the interactive session, another guessing task would follow. Analysis of the movements revealed higher movement synchrony between participants and the avatar embodying the experimenter that they associated with abstract concepts. The finding suggests that abstract concepts, likely because of their higher difficulty, elicit more interactive and pro-social behaviours: Because participants anticipate that they will need more support from other people to perform the task with abstract concepts, they tend to be more synchronous with them. The authors interpret the results by referring to the social metacognition principle (Borghi et al., 2018a, 2018b). These results pertain to the higher difficulty to infer abstract concepts from pictures; it would be important to replicate and extend this finding to other kinds of processing tasks.

Introducing another interactive methodology, Villani et al., (2021a), (2021b), under review) performed an online study in which they simulated an interaction among participants. Participants were presented with written sentences involving different kinds of concrete and abstract concepts (natural kinds, artefacts, and food; emotional and mental state concepts; philosophical and spiritual concepts; and physical spatial, temporal, and quantitative concepts) (e.g*. I made a cake*; *I thought about destiny*). They were invited to react to them as if engaging in a written conversation with a friend. Once the produced texts were coded, Bayesian statistics were applied to determine the features characterising the content of the different concepts, but also the interactive dynamics they elicited. Abstract concepts were associated with stronger uncertainty in interpreting the meaning and consequently promoted more interactive behaviours: uncertainty expressions were more frequent, together with questions, particularly “why” questions; furthermore, the number of turns was higher with abstract than with concrete concepts. Of interest is also the fact that philosophical-spiritual concepts, like *belief* and *religion*, which are more abstract than the other abstract concepts, elicited more uncertainty—more repetitions, uncertainty expressions, and questions –, more interactive behaviours—more turns—and more general statements compared to other abstract concepts. This study has the limitation to focus on a simulated interaction rather than on a real one, but, together with the other studies we reviewed, it might pave the way for new ways to investigate abstract concepts.

## Conclusion

In this review, we have explored the influence of different kinds of constraints on the acquisition, representation, and use of abstract concepts. We first focussed on external constraints, posited by perception, action, language, and culture. Then we listed some inner constraints on cognition, ascribing a special to metacognition in its interaction with inner speech and to interoception. Finally, we addressed some methodological issues, stressing the importance of understanding the time course of conceptual activation and of using novel, interactive methods to investigate abstract concepts. We ended by illustrating some methodological novelties, such as the adoption of interactive methods, that might be relevant and promising for future research in the field.
